# Similarities and Distinctions in Actions of Surface-Directed and Classic Androgen Receptor Antagonists

**DOI:** 10.1371/journal.pone.0137103

**Published:** 2015-09-02

**Authors:** Ji Ho Suh, Arundhati Chattopadhyay, Douglas H. Sieglaff, Cheryl Storer Samaniego, Marc B. Cox, Paul Webb

**Affiliations:** 1 Genomic Medicine Program, Houston Methodist Research Institute, 66670 Bertner Avenue, R8-114, Houston, Texas, 77030, United States of America; 2 Border Biomedical Research Center, Dept. of Biological Sciences, University of Texas at El Paso, 500 W. University Ave., El Paso, Texas, 79902, United States of America; Hormel Institute, University of Minnesota, UNITED STATES

## Abstract

The androgen receptor (AR) surface-directed antagonist MJC13 inhibits AR function and proliferation of prostate cancer (PC) cells. These effects are related to arrest of an AR/chaperone complex in the cytoplasm. Here, we compared MJC13 and classic AR antagonists such as flutamide and bicalutamide. Microarray analysis and confirmatory qRT-PCR reveals that MJC13 and flutamide inhibit dihydrotestosterone (DHT)-dependent genes in LNCaP PC cells. Both compounds are equally effective on a genome wide basis and as effective as second generation AR antagonists (MDV3100, ARN-509) at selected genes. MJC13 inhibits AR binding to the prostate specific antigen (PSA) promoter more strongly than flutamide, consistent with different mechanisms of action. Examination of efficacy of MJC13 in conditions that reflect aspects castrate resistant prostate cancer (CRPC) reveals that it inhibits flutamide activation of an AR mutant (ART877A) that emerges during flutamide withdrawal syndrome, but displays greatly restricted gene-specific activity in 22Rv1 cells that express a constitutively active truncated AR and is inactive against glucocorticoid receptor (GR), which can co-opt androgen-dependent signaling networks in CRPC. Importantly, MJC13 inhibits AR interactions with SRC2 and β-catenin in the nucleus and, unlike flutamide, strongly inhibits amplification of AR activity obtained with transfected SRC2 and β-catenin. MJC13 also inhibits DHT and β-catenin-enhanced cell division in LNCaP cells. Thus, a surface-directed antagonist can block AR activity in some conditions in which a classic antagonist fails and may display utility in particular forms of CRPC.

## Introduction

Androgen receptor (AR) mediates growth promoting effects of androgens in prostate cancer (PC) and is a major target for therapeutics [1–4]. Androgens bind the buried ligand binding pocket (LBP) in the AR C-terminal ligand binding domain (LBD) and induce an active AR conformation able to translocate to the nucleus, bind DNA and recruit coactivators [1,4]. Classic AR antagonists such as flutamide displace androgens from the LBP and induce a poorly defined inactive conformation that is unable to recruit coactivators and, therefore, unable to activate gene expression programs required for androgen-dependent PC growth [5,6]. Unfortunately, AR antagonists often become ineffective during extended use, and PC recurs in castrate resistant form (CRPC), which is dependent on AR but exhibits reduced requirement for androgens and is resistant to antagonists [1–3,5,6]. Suggested reasons for reactivation of AR in low androgen/antiandrogen environments include local production of androgenic ligands [7] and emergence of AR mutants with altered response to ligand [8]. Additionally, glucocorticoid receptor (GR)-dependent pathways can activate CRPC growth under conditions in which AR activity is suppressed [9]. CRPC may also be related to amplification of AR signaling caused by overexpression of AR itself, or AR coactivators, such as the steroid receptor coactivators (SRCs) [1–6]. Finally, AR activity could be potentiated by alternate AR interacting proteins, including the wnt pathway target β-catenin [10–13], which bind to AR at a distinct site from SRCs [14–16].

Effective blockade of AR action in CRPC should inhibit disease recurrence and extend patient survival [5] and several new approaches to inhibition of AR action in CRPC emerged in recent years. More effective AR antagonists with improved affinity are now available [17,18]. Additionally, the Cyp17 inhibitor Abiraterone is used to inhibit synthesis of low levels of androgens that can reactivate AR in CRPC [19]. Our groups, and others, explored the feasibility of a different route of AR inhibition, development of ligands that bind key AR/protein interaction surfaces to block coactivator recruitment [20–23]. In principle, this class of compounds could inhibit AR activity in early androgen-dependent PC and CRPC. We used screening approaches to identify surface interacting ligands that inhibit AR/coactivator interaction *in vitro* and AR activity in cultured cells. X-ray screening revealed that useful ligands clustered in a novel AR surface, BF-3, which comprises a shallow cleft formed between helices (H) 1 and 9 and the H3–H4 loop [20]. BF-3 is subject to natural mutations in PC and also in androgen insensitivity syndrome and targeted mutation of BF-3 residues confirm that it is required for AR activity [20]. Further, BF-3 may constitute a multifunctional protein interaction surface. BF-3 binds the chaperone Bag-1L through a duplicated N-terminal GARRPR motif [24]. In addition, our studies indicate that potentiation of AR activity by the chaperone FKBP52 (52Kd FK506 binding protein) requires BF-3 and β-catenin cooperates with FKBP52 to enhance AR activity through BF-3 [25]. Although BF-3 is distant from the classic coactivator binding surface activation function 2 (AF-2), which binds SRCs via LxxLL motifs and AR-specific coactivators via FxxLF motifs, ligand binding to BF-3 may also inhibit AF-2 via indirect allosteric effects [20,26]. Thus, BF-3 binding compounds should block AR interactions with a subset of interacting proteins and indirectly inhibit AF-2 and therefore modulate AR activity in a distinct way from classic AR antagonists, which work solely by inducing suboptimal AF-2 conformations.

The AR inhibitor MJC13 blocks FKBP52-stimulated AR activity in yeast and cell culture but does not interact with FKBP52 and was unable to displace androgens from the AR pocket [27]. Instead, several lines of evidence indicate that MJC13 interacts with BF-3 and acts as an AR surface-directed antagonist. Further, unlike classic AR antagonists, MJC13 promotes arrest of the AR-FKBP52-Hsp90 complex in the cytoplasm, reducing rates of AR nuclear translocation. In this study, we set out to compare efficacy of MJC13 and the classic AR antagonist flutamide. Both compounds modulate endogenous gene expression in PC cells in similar fashion despite different mechanisms of action. However, MJC13 inhibits AR activity in some conditions in which flutamide fails. We therefore suggest that MJC13 and similar compounds could provide advantages over standard therapies for some forms of CRPC.

## Materials and Methods

### Plasmids and Reagents

Mammalian expression vectors for human AR, SRC-2, MMTV-luc, human β-catenin, Gal4-AR LBD and Gal4-tk-luc were described previously [14,27,28]. ARE-luc was provided by Dr. Keesook Lee (Chonnam National University, South Korea). Mammalian expression vector for human AR T877A mutant and Gal4-AR T877A mutant were created by targeted mutagenesis (Stratagene). Flutamide was purchased from Sigma Aldrich. MDV3100, ARN-509 and bicalutamide were gifts from Dr. Robert Fletterick, Department of Biochemistry, UC San Francisco).

### Cell Culture and Transfection

HEK293T cells (purchased from ATCC) were maintained in Dulbecco's modified Eagle's medium (DMEM) containing phenol red and supplemented with 10% fetal bovine serum. LNCaP cells (purchased from the ATCC) and 22RV1 cells (purchased from ATCC by Dr. Anders Strom, University of Houston) were maintained in RPMI 1640 containing phenol red and supplemented with L-glutamine and 10% fetal bovine serum. For luciferase assays, cells were switched to medium with commercial charcoal stripped serum (Sigma) one day prior to transfection and were maintained in this medium after hormone induction. Cells were transfected using Fugene HD Transfection reagent (Roche) and luciferase assays performed as described [29]. Luciferase activities were normalized to lacZ expression. Quantities of expression vectors and reporter genes used in each experiment are listed individually in figure legends.

### Gene Expression Analysis

LNCaP cells were treated +/- DHT, +/- Flutamide, and +/- MJC-13 at indicated concentrations for 24h. Microarray hybridizations were performed as described [29]. Arrays were scanned using iScan Reader. Unmodified microarray data was obtained from GenomeStudio, subsequently background-subtracted and quantile-normalized using the lumi package [30] and analyzed with the limma package [31] within R [32]. Real-time PCR was performed as described previously [29], using the Roche LightCycler 480 II and SYBR Green Mastermix (Roche). Primers span exon-exon boundaries to preclude amplification of gDNA, the sequences of which are available upon request. Relative mRNA levels were calculated by the comparative cycle threshold method normalized to GAPDH as the internal control. Of note, GAPDH mRNA levels were not affected by ligand or chemicals in current experiments. Data was deposited publicly; GEO submissions GSE65562, NCBI tracking system #17251833.

### Chromatin Immunoprecipitation

LNCaP cells were treated +/- 1nM DHT, +/- 1μM Flutamide, or +/- 30μM MJC-13 for 16h. ChIP was performed by SimpleChip Enzymatic Chromatin IP Kit (Cell Signaling) according to manufacturer’s instructions. 10% (v/v) of the supernatant was saved as ‘input’ chromatin prior to immunoprecipitation. Anti-AR antibody (Santa Cruz Biotechnology) was used for immunoprecipitation. Immunoprecipitated DNA and input-sheared DNA were subjected to PCR using primer pair (5'-GCCTGGATCTGAGAGAGATATCATC-3'; 5’-ACACCTTTTTTTTTCTGGATTGTTG-3’), which amplify regions spanning the PSA androgen response element (ARE). IgG was used as an immunoprecipitation control.

### Immunoprecipitation and Western Blot

Coimmunoprecipitations were performed from extracts of LNCaP Cells or HEK293T cells as described using 2 μg of anti-AR or anti-Gal4 antibodies and protein A/G agarose bead (all Santa Cruz biotechnology). Cells were treated +/- hormones and antagonists as mentioned above and described in individual Figure Legends. Proteins were subjected to Western blot analysis with anti-AR (Santa Cruz Biotechnology), anti-SRC-2 (Bethyl Laboratories), anti-β-catenin (Santa Cruz Biotechnology, Cell signaling), and anti-GAPDH (Cell Signaling) and detected with an ECL kit (Amersham Pharmacia).

### Adenovirus Infection and Cell Proliferation Assay

Adenovirus that expresses mouse wild type β-catenin was purchased from Applied Biological Materials. Cell proliferation was assessed by XTT Cell Viability Kit (Cell Signaling) according to manufacturer’s instructions. LNCaP cells were plated in 96-well plates at 10^4^cells/well. Cells were infected with adenovirus expressing β-catenin (Ad-β-catenin) or null adenovirus (Ad-Null) at multiplicity of infection = 50, maintained in normal culture media with steroid depleted serum and treated with 1nM DHT, 1μM Flutamide, 1μM bicalutamide or 30μM MJC13 for 24 h. After 24 h, XTT solution was added to each well and cells were incubated for 4 h at 37°C, the absorbance was measured using an ELISA reader at a wavelength of 450 nm.

### Statistical Analysis

All results are means ± SD. Statistical analysis was performed using GraphPad Prism software (GraphPad Inc., San Diego, CA). Comparisons of groups were performed using ANOVA followed by a post hoc multiple comparisons test. P ≤ 0.05 was considered statistically significant. All experiments were performed at least three times. Throughout the manuscript, the following convention was used to denote levels of statistical significance ***,P < 0.001; **,P < 0.01; *,P < 0.05.

## Results

### MJC13 Inhibits an AR-Dependent Reporter as Efficiently as Flutamide

**Fig 1:**
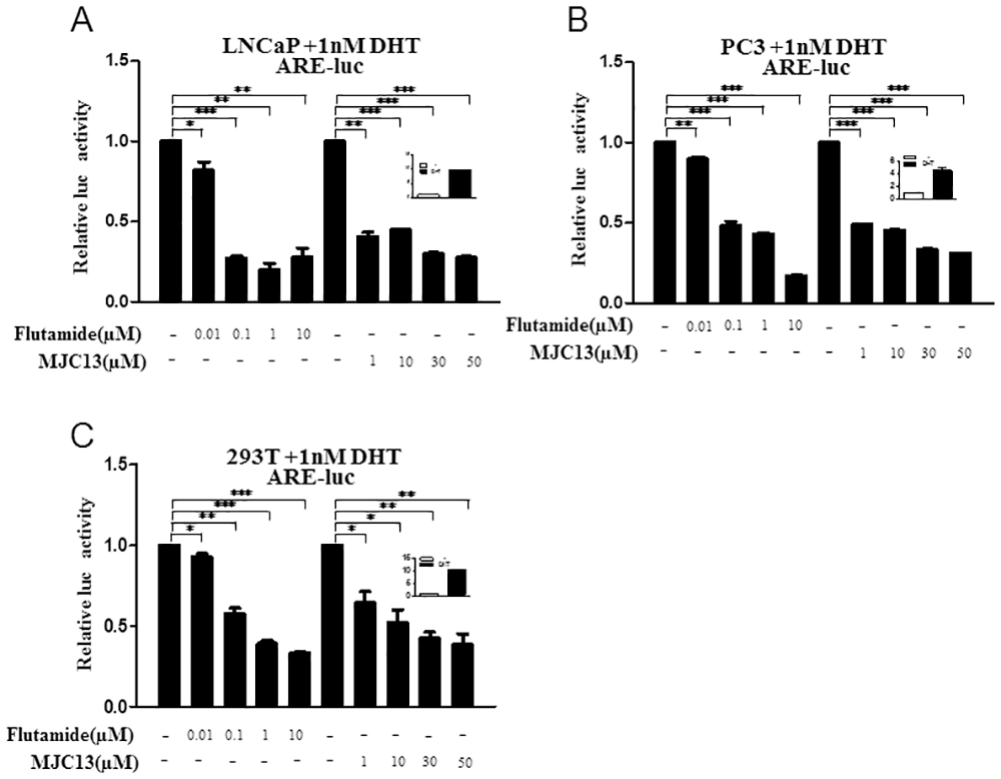
MJC13 inhibits AR transactivation. Luciferase activity measured in (A) LNCaP cells transfected with an ARE-luc reporter or (B and C) PC3 and HEK293T cells transfected with an expression vector for AR and the ARE-Luc reporter. Cells were treated with DHT (1nM) +/- increasing doses of Flutamide or MJC13 overnight. Data are representative of at least three independent experiments with similar results. All values represent mean ± SD of duplicate samples. Symbols denoting statistical significance in this figure and ensuing figures are explained in Materials and Methods. The insets show DHT response (black bars) versus vehicle control (arbitrarily set at 1.0; white bars) in each of these experimental conditions. Transfections in Fig 1A employed 200ng of ARE-luc, 100ng of b-gal whereas those in Fig 1B and 1C employed 200ng of ARE-luc, 100ng of AR expression vector and 100ng of β-gal.

We first compared effects of increasing doses of flutamide and MJC13 on androgen (DHT)-dependent reporter genes in the presence of 1nM DHT in prostate cancer and non-prostate cell lines. Representative results with a minimal ARE responsive reporter gene are shown in Fig 1. Both ligands repressed DHT (1nM)-dependent induction of ARE-regulated reporters in LNCaP prostate cancer cells that express endogenous ARs (Fig 1A) and in PC3 and HEK293T cells transfected with an AR expression vector (Fig 1B and 1C). Fold DHT inductions are shown in insets and were, respectively, 10-fold in LNCaP, 4-fold in PC3 and 10-fold in HEK293T cells. Analysis of dose responses revealed that both compounds strongly inhibited DHT-dependent activation obtained in the presence of endogenous ARs in LNCaP cells (Fig 1A). Maximal inhibitory responses were obtained with 1μM flutamide and 30μM MJC13. While MJC13 consistently appeared less potent than flutamide or other antagonists employed in this study it was equally effective at highest doses. Both AR antagonists also repressed basal ARE-dependent reporter activity in LNCaP cells in the absence of DHT (S1 Fig). All experiments in this study were performed in steroid-depleted charcoal stripped serum. Thus, this result implies that AR is weakly active in the absence of exogenous DHT in LNCaP cells in these conditions and that this activity is repressed by flutamide and MJC13 (Discussion). The two antagonists were also equally effective, but slightly less potent, in transient transfections in PC3 and HEK293T cells (Fig 1B and 1C). In this case, however, 1μM flutamide and 30μM MJC13 were still sufficient to obtain maximal or near maximal repression of DHT-dependent activity. As seen in LNCaP, both ligands weakly repressed basal reporter activity in the presence of transfected AR and the absence of DHT (not shown). Similar results were also obtained with another androgen-dependent reporter, MMTV-Luc (not shown). Thus, flutamide and MJC13 inhibit ARE driven reporters in several cell backgrounds in a dose dependent fashion and are equally effective at highest doses. We selected concentrations of antagonists (1μM flutamide, 30μM MJC13) that yielded maximal inhibition of responses obtained with 1nM DHT for future experiments.

### MJC13 and Flutamide Inhibit DHT Responsive Genes in LNCaP

**Fig 2:**
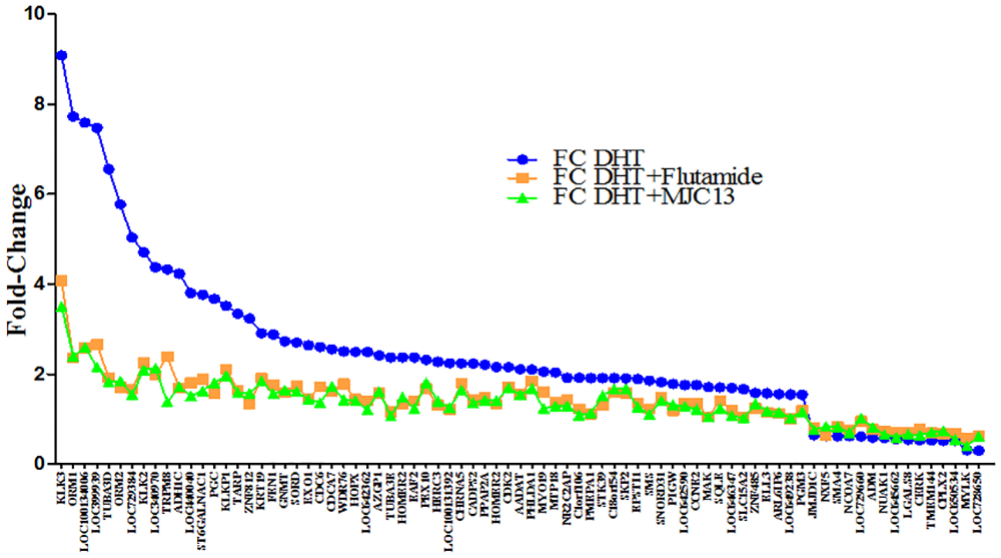
AR target genes are inhibited by MJC13 and Flutamide. Graph representing results of array analysis performed on LNCaP cells treated for 24 hours +/- DHT (1nM), +/- Flutamide (1μM), or +/- MJC13 (30μM) and displaying probe sets in which DHT response is inhibited by MJC13 and Flutamide. The first line (blue) represents DHT responses obtained in the presence of DMSO, second line (orange) represents DHT responses obtained in the presence of Flutamide. The third line (green) represents DHT responses obtained in the presence of MJC13.

**Fig 3:**
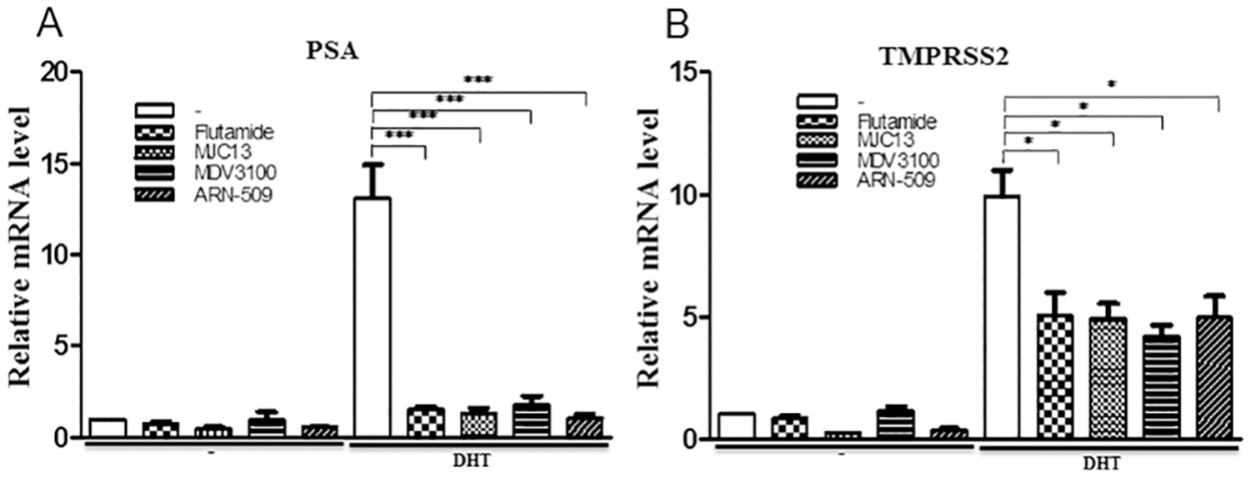
MJC13 and Classic AR antagonists display similar patterns of AR target genes inhibition. qPCR analysis of LNCaP cells extracts treated +/- DHT (1nM), +/- Flutamide (1μM), ARN-509, MDV3100 (10μM) or +/- MJC13 (30μM) for 24 hours. The data are representative of at least three independent experiments. All values represent the mean ± SD of triplicate samples. **(A)** PSA gene. **(B)** TMPRSS2 gene.

We next determined effects of both AR antagonists on the endogenous androgen regulated gene program using microarray analysis in LNCaP cells (Fig 2). Analysis of effects of aforementioned doses of flutamide and MJC13 revealed widespread inhibitory effects on genes that displayed changed expression in the presence of 1nM DHT (Fig 2). Both ligands suppressed all gene expression changes that were observed in response to DHT treatment and, further, displayed remarkably similar effects on a gene by gene basis. We verified that flutamide and MJC13 indeed displayed similar actions at selected DHT-induced genes by qRT-PCR and also compared effects of both compounds with the second generation AR antagonists MDV3100 and ARN-509 [17,18]. We found that MJC13 was equally effective as all three classic AR antagonists at suppressing DHT response at the PSA gene (Fig 3A). We observed similar effects at TMPRSS2 [33]; a gene that is a frequent target for chromosomal translocation in prostate cancer but did not meet fold cut-offs for DHT response in our analysis (Fig 3B). We also confirmed that MJC13 suppressed DHT response as efficiently as flutamide at several other DHT regulated genes that were flagged on the array (including PGC-1α, ORM 1 and 2 and WDR76 and KLK2, S2A–S2E Fig). We also detected one case in which MJC13 preferentially suppressed DHT response relative to flutamide, at the TRPM8 gene (S2F Fig).

**Fig 4:**
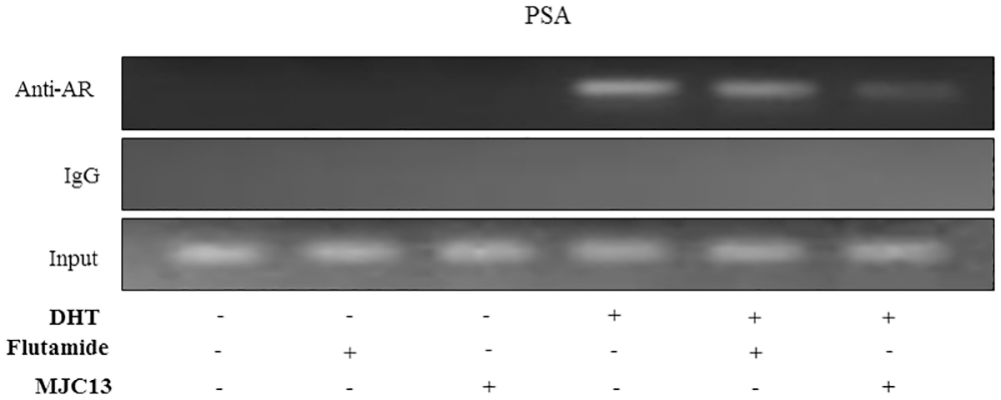
MJC13 suppresses AR recruitment to an ARE. ChIP assays performed in LNCaP cells treated +/- DHT (1nM), +/- Flutamide (1μM), or +/- MJC13 (30μM) for 16 hours. Antibodies used for immunoprecipitation were AR or IgG control. 10% (v/v) of the supernatant was represented as ‘input’ chromatin prior to immunoprecipitation by antibodies.

Given strong similarities between actions of flutamide and MJC13 on DHT response in LNCaP, we verified that the ligands act in different ways. ChIP analysis of AR binding to the PSA ARE region (Fig 4) revealed that flutamide treatment only resulted in weak inhibition of DHT-dependent AR binding to the ARE, consistent with the notion that it inhibits AR activity via changed coregulator recruitment [34]. By contrast, MJC13 treatment resulted in greatly reduced AR binding, consistent with suggestions that this ligand inhibits AR activity in a different mode from flutamide. We were not able to detect AR binding to the promoter in the absence of DHT.

### Flutamide and MJC13 Weakly Influence Basal AR-Target Gene Expression

Since we observed inhibitory effects of AR antagonists on basal ARE-dependent reporter gene activity in LNCaP cells (S1 Fig), we applied microarray analysis to assess effects of flutamide and MJC13 on endogenous genes in the absence of DHT on a genome wide level. The data are presented in S3 Fig and indicate that neither antagonist elicited strong effects on DHT regulated genes. Attempts to verify small changes seen with flutamide alone with qRT-PCR analysis were mostly unsuccessful. In a few cases, we found that both AR antagonists displayed weak inhibitory effects on basal expression of highly DHT-responsive genes and, here, MJC13 effects were usually more prominent (see Fig 3A and 3B, S2F Fig). Thus, the two antagonists do not exert large effects on endogenous gene expression in LNCaP in their own right and MJC13 inhibits basal activity of some AR-target genes (see Discussion). While this differs from expectations that flutamide displays weak partial agonist activity in LNCaP cells in the absence of androgens, lack of flutamide partial agonism in LNCaP is a common feature of many previous studies (see Discussion for citations and explanation).

### MJC13 Actions Differ from Flutamide in the Presence of Mutant ARs

**Fig 5:**
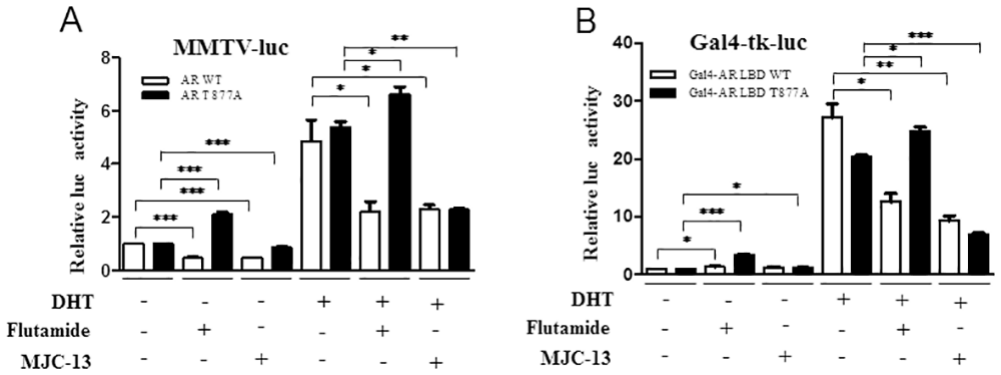
MJC13 inhibits a flutamide resistant AR mutant. Luciferase activity measured in HEK293T cells transfected with expression vectors for AR WT and AR T877A and an androgen-dependent MMTV-Luc reporter (A) or Gal4-AR LBD and Gal4-AR T877A and a GAL4 responsive reporter gene (B) and treated +/- DHT (1nM), +/- Flutamide (1μM), or +/- MJC13 (30μM) overnight, as in Fig 1. All data are representative of at least three independent experiments with similar results. All values represent the mean ± SD of duplicate samples. Transfections in Fig 5A employed 200ng of MMTV-luc, 100ng of AR or 100ng of AR T877A expression vectors and 100ng of β-gal whereas those in Fig 5B employed 300ng of Gal4-tk-luc, 200ng of Gal4-AR LBD or 200ng of Gal4-AR LBD T877A and 100ng of β-gal.

LNCaP cells express an AR mutant (T877A) that has been reported to display partial agonist activity in the presence of flutamide [17]. The ART877A mutant emerges in so-called “flutamide withdrawal syndrome”; characterized by tumor regression after withdrawal of flutamide [17]. This effect is thought to reflect loss of emergent tumor promoting agonist actions of flutamide through the mutant AR when flutamide is withdrawn. In light of this finding, we were surprised by the fact that flutamide and MJC13 inhibited basal reporter activity (S1 Fig), displayed similar inhibitory effects on DHT-regulated gene expression (Fig 2) and induced few genes in the absence of hormone (S3 Fig). We therefore set out to compare activity of MJC13 and flutamide in transfection assays in in HEK293T cells in which only wild type AR or ART877A mutant was supplied. As expected, flutamide weakly induced MMTV-LUC in the presence of AR T877A (Fig 5A, left panels); this response was comparable to previous assessments of AR T877A activity in similar conditions, see [35]. In parallel, flutamide also failed to efficiently inhibit DHT activation mediated by ART877A (Fig 5A, right panels). By contrast, MJC13 did not activate transcription in the presence of ART877A and was an effective inhibitor of DHT response in the presence of wild type AR and ART877A. Qualitatively similar responses were observed with GAL4-fusions that express wild type AR LBD or its T877A derivative (Fig 5B). Thus, MJC13 inhibits activity of this flutamide-resistant AR mutant when it is assessed in transient transfections.

**Fig 6:**
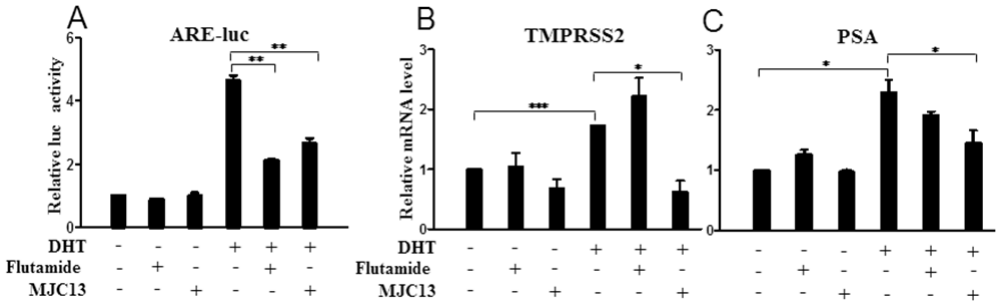
Effects of AR antagonists are blunted in 22RV1 cells. (A) Luciferase activity measured in 22RV1 cells transfected with ARE-luc reporter and treated +/- DHT (1nM), +/- Flutamide (1μM), or +/- MJC13 (30μM) overnight, with quantities of plasmids as in Fig 1A. All data are representative of at least three independent experiments with similar results. All values represent the mean ± SD of duplicate samples. (B-C) qPCR analysis of 22RV1 cells extracts treated +/- DHT (1nM), +/- Flutamide (1μM), or +/- MJC13 (30μM) for 24 hours. The data are representative of at least three independent experiments. All values represent the mean ± SD of triplicate samples. Genes are TMPRSS2 (B) and PSA (C).

We next compared activities of both compounds in 22RV1 cells, which express wild type AR and a truncated AR variant with constitutive activity [36]. We observed modest DHT activation of an ARE responsive reporter in this cell line and this effect was suppressed by flutamide and MJC13 (Fig 6A). We also observed MJC13-specific suppression of DHT response at the TMPRSS2 gene (Fig 6B). However, MJC13 failed to exhibit strong suppressive effects at other DHT-induced genes, including PSA (Fig 6C) and others (S4 Fig) in this cell background. Further, we observed that several genes which respond to DHT in LNCaP (ORM2, WDR76, KLK2 and TRPM8) did not exhibit DHT response in 22RV1 and, here, MJC13 had no effect (not shown). This suggests that the presence of the truncated variant AR alters the spectrum of androgen responsive and curtails a large proportion of MJC13 suppressive effects.

**Fig 7:**
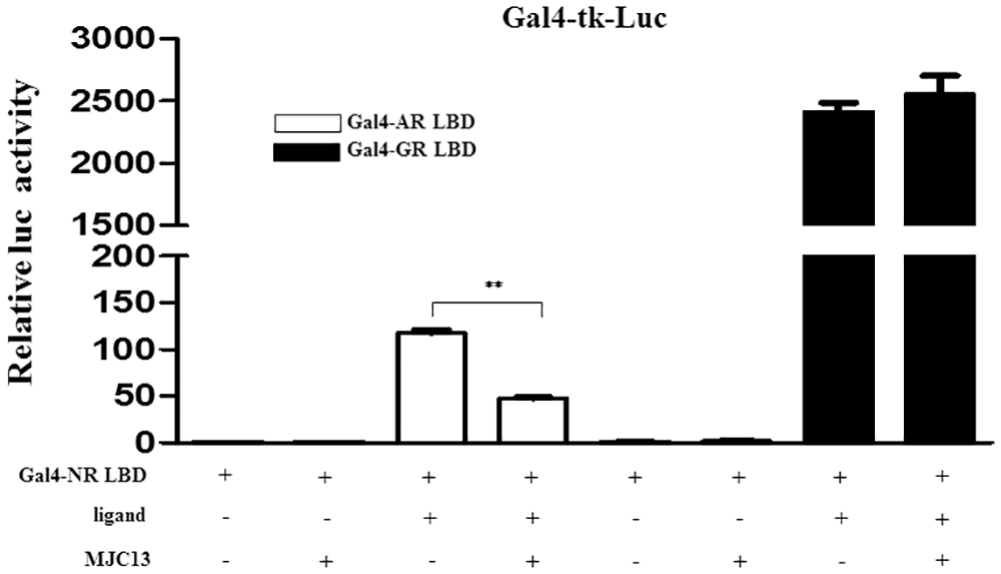
MJC13 does not influence the GR LBD. Luciferase activity measured in HEK293T cells transfected with Gal4-tk-luc reporter, Gal4-AR LBD or Gal4-GR LBD and treated +/- DHT (1nM) for AR, or +/- Dexamethasone for GR (1nM), and +/- MJC13 (30μM). All data are representative of at least three independent experiments with similar results. All values represent the mean ± SD of duplicate samples. Transfections employed 400ng of Gal4-tk-luc, 200ng of Gal4-AR LBD or 200ng of Gal4-GR LBD and 100ng of β-gal.

Despite the fact that very high doses of MJC13 have previously been shown to inhibit GR in a yeast system [27], we found that MJC13 responses were AR-selective in mammalian cells (Fig 7). While 30μM MJC13 inhibited DHT response mediated by a GAL4-AR LBD fusion, similar doses did not affect dexamethasone responses mediated by an equivalent GAL4-GR LBD fusion in HEK293T cells. Attempts to increase MJC13 dose failed to elicit GR inhibition (not shown). Thus, we suspect that it is unlikely that MJC13 will not be useful for inhibition of glucocorticoid responsive CRPC at doses used in this study [9].

### MJC13 Inhibits AR Interactions with SRC2 and β-catenin

**Fig 8:**
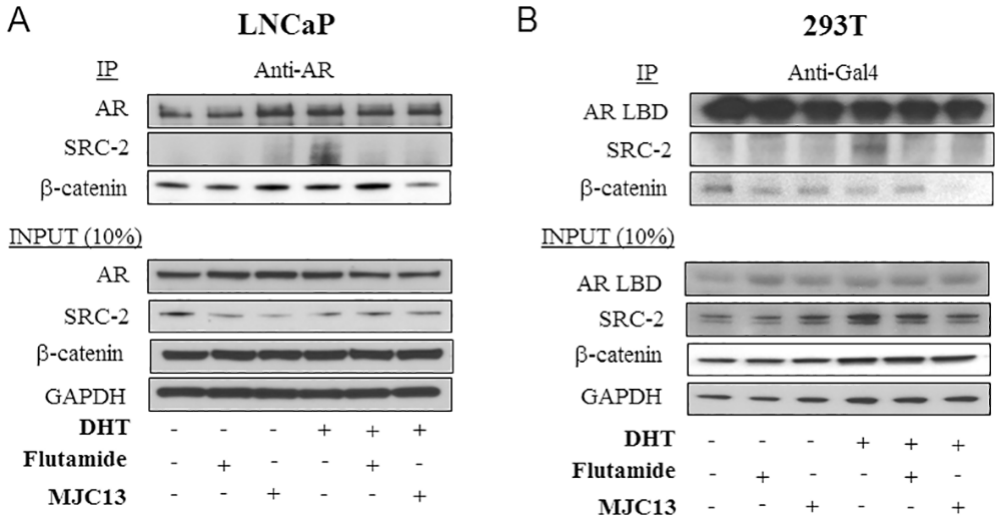
MJC13 blocks AR interaction with coactivators. (A) Co-immunoprecipitations from extracts of LNCaP cells treated +/- DHT (1nM), +/- flutamide (1μM), or +/- MJC13 (30μM) for 12 h. AR antibody was used for immunoprecipitation and antibody used for western analysis is indicated at the left hand side. Panels below represent western blots of input proteins or GAPDH loading control. (B) Co-immunoprecipitation assay from extracts of HEK293T cells cotransfected with plasmids of Gal4-AR LBD fusion and SRC-2 or β-catenin. AR LBD was immunoprecipitated with an anti-Gal4 antibody in lysates from cells treated +/- DHT, +/- Flutamide or +/- MJC13 as above. Antibody used for western analysis is indicated at the left hand side. Panels below represent western blots of input proteins or GAPDH loading control.

We next compared abilities of flutamide and MJC13 to inhibit AR/coactivator interactions. We performed co-immunoprecipitations from LNCaP cells with anti-AR antibody in the presence and absence of DHT and antagonists. SRC2 associated with AR in the presence of DHT (Fig 8A). While flutamide and MJC13 did not induce AR/SRC2 association when used alone, both compounds blocked DHT-dependent AR/SRC2 interaction [34]. Unlike SRC2, β-catenin coprecipitated with AR in the presence and absence of DHT (Fig 8A). This binding event was unaffected by antagonists in the absence of DHT, but MJC13 specifically inhibited AR/β-catenin binding in the presence of DHT, consistent with predictions of a distinct binding mode for this ligand [27]. Similar results were obtained in co-immunoprecipitations from HEK293T cells transfected with GAL4-AR LBD and coactivators. Again, flutamide and MJC13 blocked DHT-dependent AR/SRC2 association and MJC13 selectively inhibited hormone-independent AR LBD/β-catenin interactions (Fig 8B).

**Fig 9:**
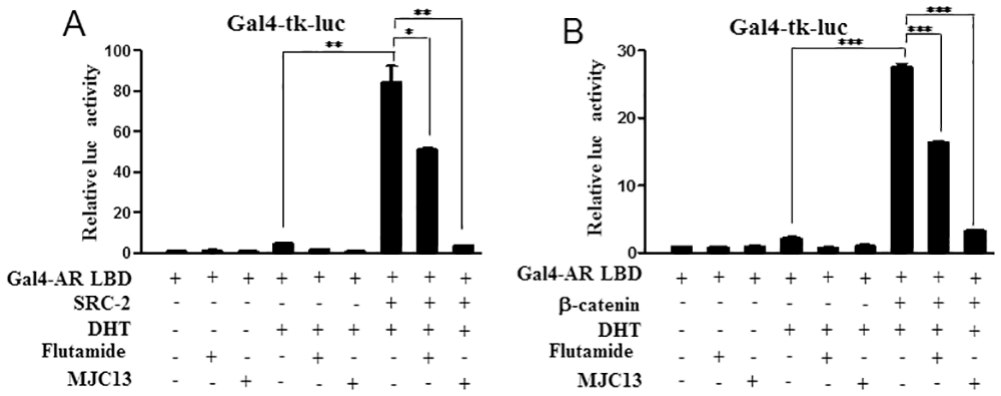
MJC13 inhibits coactivator-enhanced AR activity. Results of luciferase assays performed in HEK293T cells transfected with plasmids of Gal4-AR LBD fusion and SRC-2 (A) or β-catenin (B) and treated +/- DHT (1nM) and +/- Flutamide (1μM) or +/- MJC13 (30μM) overnight. The data are representative of at least three independent experiments. All values represent the mean ± SD of duplicate samples. Transfections employed 100ng of Gal4-tk-luc, 50ng of Gal4-AR LBD, 400ng of SRC-2, 100ng of β-gal in Fig 9A and 100ng of Gal4-tk-luc, 50ng of Gal4-AR LBD, 400ng of b-catenin, 100ng of β-gal in Fig 9B.

Since SRC2 overexpression and β-catenin activation may occur in CRPC, we determined whether overexpression of either AR coactivator influences flutamide or MJC13 sensitivity. To do this, we performed transient transfections with relatively low amounts of AR expression vectors to highlight coactivator effects, as previously described [37]. As expected, DHT induction obtained with a transfected GAL-AR LBD fusion protein was blocked by flutamide and MJC13 (Fig 9A and 9B). Further, co-transfection of SRC2 or β-catenin strongly potentiated DHT response. While both coactivators partially abrogated the ability of flutamide to suppress DHT induction, MJC13 retained the ability to suppress DHT response in the same conditions. Similar results were also obtained in mammalian two-hybrid experiments that used GAL4-AR LBD and β-catenin fused to the Vp16 activation domain (not shown). Thus, MJC13 inhibits AR activity in a manner that is insensitive to SRC2 and β-catenin overexpression.

### MJC13 Inhibits DHT and β-catenin Dependent LNCaP Cell Growth

**Fig 10:**
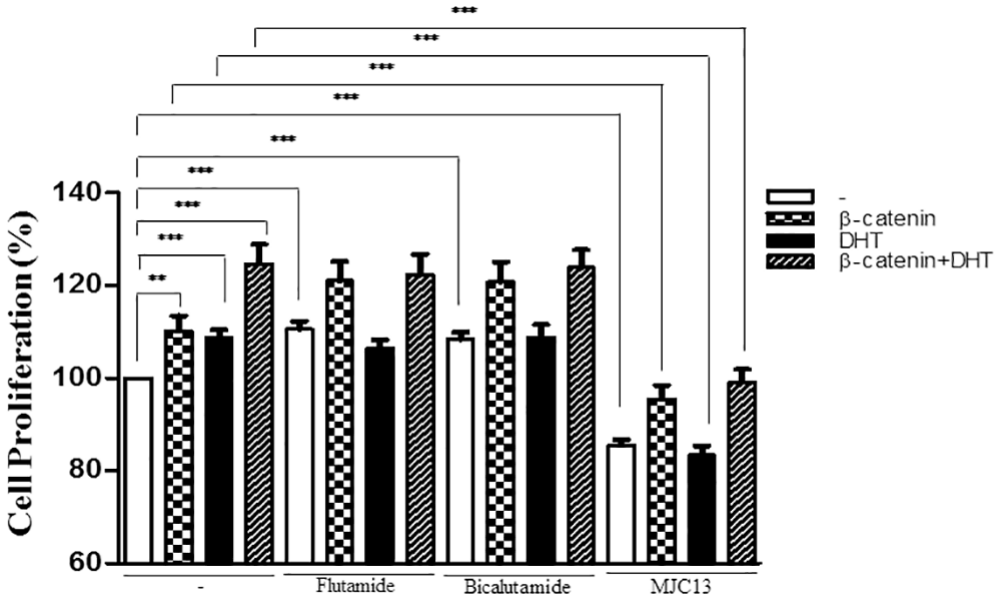
MJC13 inhibits β-catenin-induced LNCaP cell proliferation. LNCaP cells were infected with adenovirus expressing β-catenin (Ad-β-catenin) or null adenovirus (Ad-Null) at multiplicity of infection = 50, and maintained in normal media with steroid depleted serum. Cells were treated +/- DHT (1nM) +/- 1μM Flutamide, Bicalutamide or 30μM MJC13 for 24 h. The absorbance was measured using an ELISA reader at a wavelength of 450 nm. All data are representative of at least three independent experiments with similar results. Values represent the mean ± SD of triplicate samples. *** P < 0.001; ** P < 0.01; * P < 0.05. The shading scheme is as follows: White bars, adenovirus control, squared bars, Ad-β-catenin, black bars, adenovirus control + DHT, striped bars, Ad-β-catenin + DHT.

Finally, we examined effects of MJC13 on androgen-dependent LNCaP cell growth +/- β-catenin (Fig 10). As was the case in transfection and gene expression analysis, all treatments were carried out in steroid-depleted charcoal stripped serum. In basal conditions, adenovirus mediated β-catenin overexpression or DHT treatment enhanced AR-dependent LNCaP proliferation by about 10% during a 24 hour treatment and the combination of both treatments increased cell growth by more than 20%. Both flutamide and bicalutamide enhanced proliferation in their own right, consistent with documented agonist effects of both ligands on cell proliferation in the presence of ART877A in this cell-context [18, 27], whereas MJC13 inhibited proliferation by around 15%, as seen in reference 27. Adenovirus expressed β-catenin further enhanced proliferation in the presence of flutamide and bicalutamide. By contrast, LNCaP cell proliferation rates were suppressed in the presence of MJC13, irrespective of the presence or absence of DHT. Although β-catenin did enhance proliferation in the presence of MJC13 relative to levels seen with control virus, overall proliferation remained much lower than that seen in the absence of antagonist. Thus, MJC13 can inhibit basal, DHT and β-catenin stimulated proliferation in this AR-dependent prostate cancer cell line and MJC13 actions differ from those of flutamide or bicalutamide.

## Discussion

We showed here that two different classes of AR antagonists display similar effects on androgen responsive genes in LNCaP cells in the presence of DHT. MJC13, which acts at the AR surface, inhibits DHT response at standard ARE-dependent reporters and androgen responsive genes and, further, inhibitory effects are indistinguishable from the classic AR antagonist flutamide. There were gene-specific variations in blockade of DHT response. For example, flutamide and MJC13 only inhibit DHT response at the PSA gene by about 60% but block the highly induced ORM1 and ORM2 genes by close to 100%. We also observed one case in which MJC13 was moderately more effective than flutamide (TRPM8). Notably, however, we did not detect any DHT-responsive genes that were unaffected by either of the two antagonists. Two lines of evidence presented in this paper confirm previous suggestions that MJC13 and flutamide work in different ways. Flutamide did not display significant effects on DHT-dependent AR binding to the PSA ARE whereas MJC13 almost completely inhibited AR binding. Further, both compounds blocked AR interactions with SRC2, but MJC13 was uniquely able to inhibit AR interactions with β-catenin in the presence of DHT. Thus, optimal doses of flutamide and MJC13 result in effective and specific inhibition of AR activity and effects of flutamide and MJC13 are almost indistinguishable at the level of highly DHT induced genes.

Our results differ from a previous comparison of a classic thyroid hormone (TH) receptor (TR) antagonist (NH-3) and a compound that blocks TR activity via covalent attachment to TR AF-2 (SJ-AK) [38]. Here, both compounds inhibited a range of TH responses. SJ-AK, however, only inhibited a subset of TH signaling network genes, implying that antagonists directed to AF-2 display distinct pharmacological properties from TR antagonists that target the LBP. We suspect that MJC13 displays widespread inhibition of AR action because it targets BF-3, essential for AR nuclear translocation and coregulator interaction, unlike SJ-AK, which targets a coactivator binding surface that may only be essential at some genes.

The two AR antagonists exhibited no major effects on gene expression in the absence of DHT. Microarray analysis revealed few statistically significant changes in gene expression in the absence of DHT and qRT-PCR analysis confirmed this impression for flutamide and also showed that MJC13 could inhibit basal activity of selected genes, such as PSA and TMPRSS2 (see Fig 3) and others (S4 Fig). We do not know why MJC13 displays gene-specific effects in the absence of DHT. We suspect that many MJC13 inhibitory actions stem from blockade of weak AR activation in the absence of hormone and could reflect the capacity of MJC13 to arrest the AR in the cytoplasm [27]. Consistent with this suggestion, we note that ARN-509, which also arrests AR in the cytoplasm and inhibits DNA binding activity [18], displays similar effects on PSA and TMPRSS2 (see Fig 3). This idea, however, may not completely explain all MJC13 actions and MJC13 could also act directly on nuclear ARs and remodel AR/coregulator complexes that are able to engage with DNA elements in the absence of exogenous hormones.

While not the main focus of this paper, our results also reveal complex effects of flutamide in LNCaP. As mentioned, LNCaP cells express an ART877A mutant that has confers the capacity to respond to flutamide as a partial agonist [17, 35]. We find, however, flutamide suppresses activities of this mutant AR at transfected reporter genes in the presence and absence of DHT in LNCaP (Fig 1 and S1 Fig) and inhibits endogenous genes in the presence of DHT (Figs 2 and 3, S2 Fig). Further, in the absence of hormone, flutamide only weakly alters gene expression, at best, and does not affect basal expression levels of most AR-target genes (S3 Fig). We verified that flutamide displayed expected partial agonist activities in transient transfections that utilized ART877A, but not wild type AR, in HEK293T cells, confirming previous results [35]. We also emphasize that, even though flutamide displays no prominent partial agonist actions through ART877A in LNCaP, we were able to confirm previous findings [35] that flutamide does enhance LNCaP cell proliferation (Fig 10).

Reasons that we do not detect flutamide-dependent induction of endogenous genes in the presence of the ART877A mutant in LNCaP are not clear but our results are not outside of the norm. Some groups have detected weak flutamide-dependent induction of selected genes, but not others, in conditions in which high levels of DHT induction are obtained see, for example, [39]. Other groups detect weak flutamide dependent induction of PSA protein accumulation [40,41]. However, many studies failed to detect flutamide effects on basal gene expression in LNCaP in the absence of DHT [40–46]. Possible explanations for our own inability, and that of others, to detect flutamide-dependent partial agonism is that the balance of flutamide-dependent coactivator and corepressor recruitment differs in different conditions [39] or that AR levels vary. It is especially puzzling that flutamide did not exert obvious effects on gene expression in our hands yet nevertheless stimulated LNCaP cell division (Fig 10). Perhaps small and generally hard to detect flutamide effects on gene expression translate into larger effects on growth when integrated over long times. It is also conceivable that partial agonist effects of flutamide are detectable at certain time points that were not assayed here and that these are sufficient to trigger LNCaP cell division. This issue clearly merits further investigation.

The fact that flutamide and MJC13 work in different ways raises the exciting possibility that surface-directed antagonists could block AR action in several conditions in which classical antagonists are ineffective. Although we were not able to detect flutamide partial agonist effects on gene expression in LNCaP, transfection assays revealed that MJC13 inhibits flutamide action through ART877A mutant [35]. This may suggest that MJC13 could be useful in flutamide withdrawal syndrome. By contrast, MJC13 is only partly effective at an ARE-dependent reporter and at one gene (TMPRSS2) in 22RV1 cells and does not affect other AR target genes in this cell background. Thus, it seems unlikely that MJC13 would be fully effective in PC cells that express constitutively active AR variants. Finally, previous studies have shown that very high doses of MJC13 can inhibit GR activity in yeast cells [27]. We found that MJC13 does not affect GR activity in transfections in mammalian cells, implying that it will not work in conditions in which GR subsumes AR signaling networks [9]. We do not know why MJC13 inhibits AR activity in mammalian cells, but is ineffective against the closely related GR in the same conditions. Our preliminary studies indicate that β-catenin does not enhance GR activity in the same systems (not shown), raising the possibility that differences in AR and GR associated cofactors could play a role in this differential effect.

Perhaps more strikingly, MJC13 remains effective in the presence of overexpressed coactivators and, notably, β-catenin. While flutamide blocks AR interactions with SRC2 in co-immunoprecipitations, SRC2 overexpression overcomes flutamide blockade. This effect is probably related to the inability of small ligands such as flutamide to induce a stable inactive AF-2 conformation, thus permitting overexpressed AR cofactors to overcome this inhibitory effect [47]. Flutamide also failed to inhibit AR interactions with β-catenin or AR potentiation by overexpressed β-catenin. By contrast, MJC13 inhibits AR interactions with both coactivators in the presence of DHT and remains effective when either coactivator is overexpressed. Indeed, we were able to confirm that DHT cooperates with β-catenin to enhance LNCaP proliferation and show that MJC13 efficiently blocks these effects, different from flutamide and bicalutamide which display agonist effects through ART877A in this context.

We do not know how MJC13 blocks AR/β-catenin interactions but emphasize that we do not think that MJC13 directly blocks AR/β-catenin interactions in the presence of DHT because we have been unable to demonstrate effects of MJC13 upon DHT-AR/β-catenin interactions in pulldowns *in vitro* (not shown). Our accompanying paper indicates that β-catenin cooperates with FKBP52 to stimulate AR and we therefore suggest that this effect may be related to preferential inhibition of DHT-dependent changes in the AR/FKBP52 complex and nuclear translocation rather than direct effects on AR/β-catenin binding [48]. In either case, the fact that an AR surface directed antagonist blocks AR activity in the presence of overexpressed β-catenin suggests that it will be interesting to determine whether such compounds could be useful in blocking AR reactivation that occur in response to wnt pathway stimulation in CRPC. While relatively high doses of MJC13 are needed to obtain maximal suppression of androgen response, suggesting that this compound is not yet ideal for treatment of PC or CRPC, we propose that optimized versions of compounds that work in this way will be useful additions to the armamentarium of treatments for PC.

## Supporting Information

S1 Fig MJC13 and Flutamide inhibit basal activity of an ARE-reporter in the absence of hormone. Results of transfection analysis showing basal activity of the ARE-Luc reporter in LNCaP Cells +/- Flutamide and MJC13. Transfections employed 200ng of ARE-luc and 100ng of β-gal.
(TIF)

S2 Fig MJC13 and Flutamide display similar patterns of AR target gene inhibition. qPCR analysis of LNCaP cells extracts treated +/- DHT, +/- Flutamide or MJC13. The data are representative of at least three independent experiments. All values represent the mean ± SD of triplicate samples. **(A-F)** PGC-1, ORM1, ORM2, WDR76, KLK2, TRPM8. Note that MJC13 displays more potent inhibitory effects at the latter two genes.
(TIF)

S3 Fig MJC13 and Flutamide do not display partial agonists at AR target genes in LNCaP. Information from microarray analysis, presented as in Fig 2 of the main text. The red trace represents fold change of DHT regulated genes in LNCaP cells. Purple and Cyan traces represent flutamide and MJC13 responses in the same conditions; note these are all close to 1 (= unchanged).
(TIF)

S4 Fig Effects of AR antagonists are blunted in 22RV1 cells. (A, B) qPCR analysis of 22RV1 cells extracts treated +/- DHT (1nM), +/- Flutamide (1μM), or +/- MJC13 (30μM) for 24 hours as in Fig 6. The data are representative of at least three independent experiments with values representing the mean ± SD of triplicate samples. Genes are PGC1 (A) and ORM1 (B). Symbols denoting statistical significance are listed in Materials and Methods.
(TIF)
