# Supplementary figures and images for: Similarities and Distinctions in Actions of Surface-Directed and Classic Androgen Receptor Antagonists
Source: PLoS One. 2015 Sep 2;10(9):e0137103. doi: 10.1371/journal.pone.0137103 (PMC4557941; doi:10.1371/journal.pone.0137103)

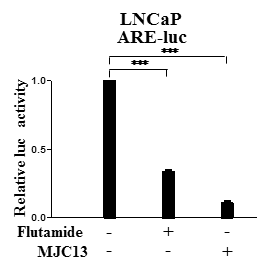

Supplement: S1 Fig — Results of transfection analysis showing basal activity of the ARE-Luc reporter in LNCaP Cells +/- Flutamide and MJC13. Transfections employed 200ng of ARE-luc and 100ng of β-gal. (TIF) [file pone.0137103.s001.tif]

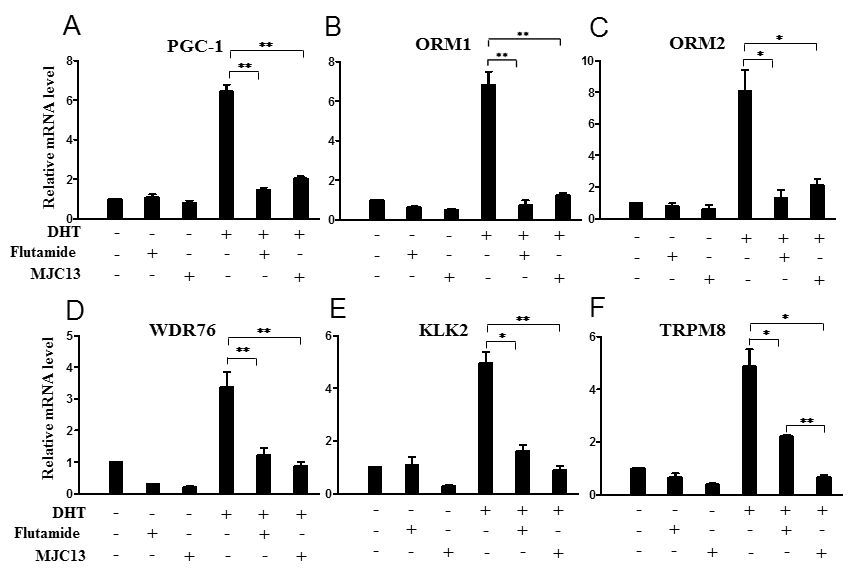

Supplement: S2 Fig — qPCR analysis of LNCaP cells extracts treated +/- DHT, +/- Flutamide or MJC13. The data are representative of at least three independent experiments. All values represent the mean ± SD of triplicate samples. (A-F) PGC-1, ORM1, ORM2, WDR76, KLK2, TRPM8. Note that MJC13 displays more potent inhibitory effects at the latter two genes. (TIF) [file pone.0137103.s002.tif]

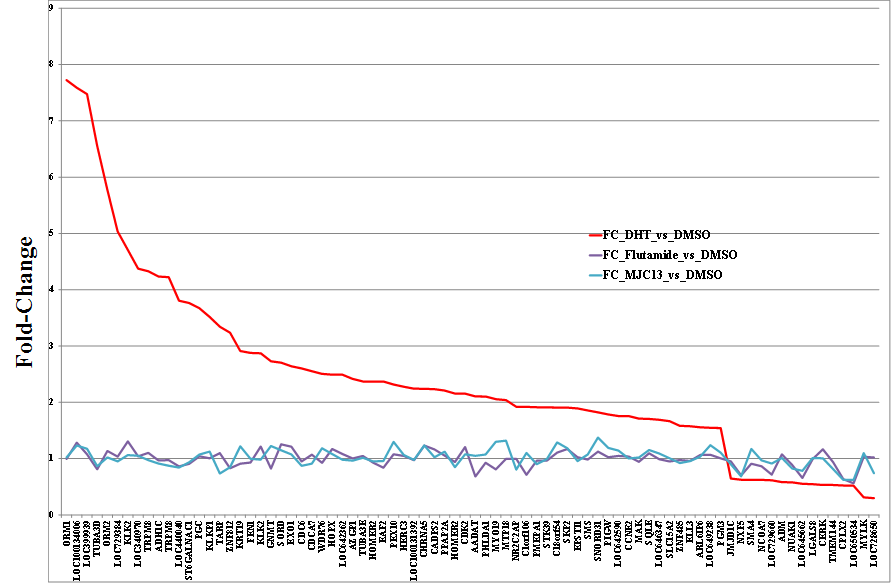

Supplement: S3 Fig — Information from microarray analysis, presented as in Fig 2 of the main text. The red trace represents fold change of DHT regulated genes in LNCaP cells. Purple and Cyan traces represent flutamide and MJC13 responses in the same conditions; note these are all close to 1 (= unchanged). (TIF) [file pone.0137103.s003.tif]

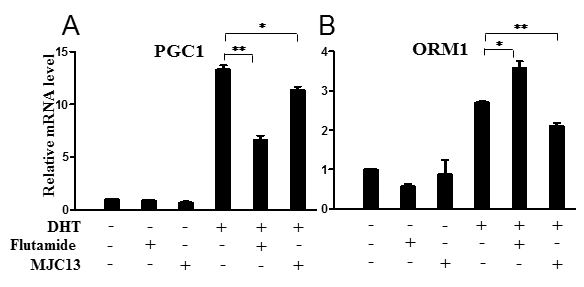

Supplement: S4 Fig — (A, B) qPCR analysis of 22RV1 cells extracts treated +/- DHT (1nM), +/- Flutamide (1μM), or +/- MJC13 (30μM) for 24 hours as in Fig 6. The data are representative of at least three independent experiments with values representing the mean ± SD of triplicate samples. Genes are PGC1 (A) and ORM1 (B). Symbols denoting statistical significance are listed in Materials and Methods. (TIF) [file pone.0137103.s004.tif]
